# Lipid Profile Management in Secondary Prevention in Spain: Data from the BDCAP Registry in Spain (LIPIDSPAIN)

**DOI:** 10.3390/jcm14176037

**Published:** 2025-08-26

**Authors:** Miguel García-Villarino, Carmen Lambert, Tomás González-Vidal, Ana Victoria García, Elsa Villa-Fernández, Claudia Lozano-Aida, Lorena Suárez-Gutiérrez, Pedro Pujante, Elías Delgado, Edelmiro Menéndez-Torre, Jessica Ares-Blanco

**Affiliations:** 1 Department of Medicine, University of Oviedo, 33003 Oviedo, Spain; garciavmiguel@uniovi.es (M.G.-V.); elsavfer@gmail.com (E.V.-F.); eliasdelga@gmail.com (E.D.); edelangot@gmail.com (E.M.-T.); jessiaresb@gmail.com (J.A.-B.); 2 Instituto de Investigación Sanitaria del Principado de Asturias (ISPA), 33011 Oviedo, Spain; tomasgonvidal@gmail.com (T.G.-V.); vctrgago@gmail.com (A.V.G.); c.lozaida@gmail.com (C.L.-A.); loresuarezgu@gmail.com (L.S.-G.); pedropujanteal@gmail.com (P.P.); 3 Instituto Universitario de Oncología del Principado de Asturias (IUOPA), 33006 Oviedo, Spain; 4 Servicio de Endocrinología y Nutrición, Hospital Universitario Central de Asturias (HUCA), 33011 Oviedo, Spain

**Keywords:** secondary prevention, lipid-lowering therapy, LDL cholesterol, cardiovascular disease, BDCAP registry

## Abstract

**Introduction**: Achieving LDL cholesterol (LDL-C) targets is critical in secondary cardiovascular prevention. Despite clinical guidelines promoting aggressive lipid-lowering strategies, many patients fail to reach recommended LDL-C levels. This study aimed to evaluate lipid profile management among secondary prevention patients in Spain using the Spanish Primary Care Clinical Database (BDCAP) registry. **Methods**: A repeated cross-sectional study was conducted using 2019–2023 data from the BDCAP. Patients with prior diagnoses of ischemic heart disease, stroke, or peripheral artery disease, and receiving lipid-lowering therapy, were included. Data on therapy type (monotherapy or combination therapy with lipid-lowering drugs), LDL-C serum levels, and demographic and socioeconomic factors were analyzed. Trends from 2019 to 2023 and regional differences were also explored. **Results**: In 2023, 1,565,429 patients received lipid-lowering drugs for secondary prevention (678.3 per 1000 attended), with higher rates in men. Combination therapy increased over time, from 88.9 to 191.1 per 1000 between 2019 and 2023. Regional disparities were notable, with treatment coverage ranging from 53.9% to 87.9%. Only 33.7% of treated patients achieved LDL-C < 70 mg/dL, and 65.6% achieved <100 mg/dL. Combination therapy was significantly more effective than monotherapy in reaching both LDL-C thresholds. **Conclusions**: Despite the growing use of combination lipid-lowering therapy, a substantial proportion of secondary prevention patients in Spain do not meet LDL-C targets. These findings highlight the need for more intensive lipid management strategies and improved adherence to clinical guidelines to optimize cardiovascular risk reduction.

## 1. Introduction

Achieving low-density lipoprotein cholesterol (LDL-C) targets is essential for secondary prevention in patients with established atherosclerotic cardiovascular disease, as aggressive LDL-C lowering significantly reduces subsequent cardiovascular events [1]. Despite this, real-world evidence indicates substantial gaps in target attainment. For instance, observational studies report that fewer than 30% of patients with ASCVD reach guideline-recommended LDL-C reductions [2]. In specific high-risk subgroups, such as those post-revascularization, achievement rates can be as low as 5%, while, in other populations, they may climb to approximately 70% [3]. Even for the stricter LDL-C threshold of <70 mg/dL, attainment remains low, with success rates documented between 33% and 35% [4]. Notably, although statin usage is relatively high (61–83%), a significant proportion of patients still do not achieve their lipid targets [3]. These findings underscore a persistent treatment gap in real-world settings and highlight the need for intensified and more tailored lipid-lowering strategies.

Most European and Spanish guidelines [5,6] recommend an LDL-C target below 70 mg/dL for patients in secondary prevention, particularly for those at extremely high cardiovascular risk [7,8,9]. In certain cases, such as patients with recurrent cardiovascular events, lower targets can be set, even below 40 mg/dL. However, in our analysis, due to the data structure of the BDCAP registry, LDL-C results were only available in predefined thresholds of <70 mg/dL and <100 mg/dL. Therefore, it was not possible to evaluate the proportion of patients achieving <55 mg/dL or <40 mg/dL, as recommended by the 2019 ESC/EAS guidelines for very high-risk patients. In addition to achieving a specific LDL cholesterol level, some guidelines also recommend a relative reduction of 50% from baseline [1]. Regarding treatment strategies, current European Society of Cardiology (ESC) and Spanish Society of Cardiology (SEC) guidelines recommend a stepwise approach, starting with high-intensity statin monotherapy (e.g., atorvastatin 40–80 mg or rosuvastatin 20–40 mg) [5,6]. If LDL-C goals are not met within 4–6 weeks, early intensification with ezetimibe is advised, and in very high-risk patients, such as those with recent acute coronary syndrome, combination therapy can be initiated upfront [5,10,11]. When targets remain unmet despite statin plus ezetimibe, PCSK9 inhibitors are recommended [5,12], while other agents (fibrates, bile acid sequestrants, bempedoic acid) may be considered in selected cases [5,13]. In Spain, atorvastatin and rosuvastatin are the most prescribed statins [14,15], often combined with ezetimibe [10]. PCSK9 inhibitor use remains limited due to cost and eligibility restrictions [12,16]. In some patients, older-generation combinations (e.g., statin + fibrate) are used to address both LDL-C and triglycerides [5,13], although evidence shows that appropriately selected combination therapy, whether with older or newer drugs, tends to outperform monotherapy in achieving stringent LDL-C targets [10,11].

One study involving 1909 participants demonstrated that only 41.3% met LDL-C targets according to the 2019 European Atherosclerosis Society/ESC guidelines [17]. Among a cohort of younger patients with acute coronary syndrome, 62.4% achieved LDL-C targets in 6 months, falling to 52.4% at one year [18]. In Albania, only 21.4% of patients met LDL-C targets after 6 months [19]

The lipid profile of the Spanish population has been a significant focus in cardiovascular disease (CVD) prevention research. While Spaniards have total cholesterol and LDL-C levels comparable to other developed countries, they tend to exhibit higher High-Density Lipoprotein cholesterol (HDL-C) levels, potentially contributing to lower CVD mortality rates [20]. However, studies involving patients treated with statins indicate suboptimal lipid control.

In the DYSIS-Spain study, 78.9% of high-risk patients had abnormalities in at least one of the three main lipid parameters (LDL-C, HDL-C, or triglycerides), while [21] 61.4% did not reach their target LDL-C levels [14]. Similarly, in primary care settings, only 4.7% of patients with ischemic heart disease achieved LDL-C levels below 100 mg/dL, while 31.3% reached levels below 130 mg/dL [22].

In patients with cerebral atherosclerotic disease, only 15% achieved LDL cholesterol targets [21]. The underutilization of combination therapies in this group suggests a need for more aggressive treatment strategies to achieve LDL cholesterol targets. There are less data published on peripheral artery disease, but similar trends are expected in achieving LDL cholesterol goals, given the lower overall rates of compliance [21].

Beyond LDL-C control, there is growing recognition of the importance of addressing atherogenic dyslipidemia, characterized by elevated triglyceride and apolipoprotein B levels, especially in the context of increasing obesity and insulin resistance. In this setting, treatment success refers to achieving a significant reduction in cholesterol levels, particularly LDL-C and other atherogenic lipoproteins. Factors associated with greater likelihood of success include participation in cardiac rehabilitation programs, having experienced a recent acute coronary event, adherence to combination lipid-lowering therapies, as well as engagement in healthy lifestyle habits such as dietary improvements and regular physical activity [17,18]. In contrast, older age and the absence of lipid-lowering treatment are associated with a lower probability of achieving cholesterol reduction [17].

These findings suggest significant room for improvement in lipid management for secondary prevention within the Spanish population. Therefore, the aim of this study is to evaluate lipid profile management in secondary prevention patients in Spain, using data from the Primary Care Clinical Database (BDCAP) registry (LIPIDSPAIN).

## 2. Material and Methods

### 2.1. Contextualization, Data Request, and Eligibility Criteria

The BDCAP is a structured and standardized dataset designed to offer a longitudinal view of healthcare delivery within the Spanish Healthcare System (SHS). It aggregates clinical data from electronic health records used in primary care. The latest update, conducted in 2023, includes information from individuals receiving care in primary care services across Spain. While the BDCAP relies on a representative sample of approximately 4.8 million electronic health records from primary care, the data utilized in this study were sourced from the Ministry of Health’s statistical portal. This platform extrapolates information to reflect the entire population covered by the SHS, enabling nationally representative estimates broken down by Autonomous Community, age, sex, and other demographic variables. Based on the extrapolated figures for 2023, a total of 46.7 million individuals were assigned to the SNS. BDCAP collects information on diagnosed health conditions, prescribed and dispensed medications, and clinical parameters, anonymized and coded using standardized classification systems such as the International Classification of Diseases (ICD9, ICD10ES) and the International Classification of Primary Care (CIAP2) for health conditions, while medications are coded using the National Code for drugs. For this study, we conducted a repeated cross-sectional analysis without interventions, utilizing the statistical portal of the Ministry of Health, which is linked to the BDCAP database. Our focus was on patients undergoing lipid-lowering therapy for secondary prevention, defined as individuals with a previous diagnosis of ischemic heart disease, stroke, or peripheral artery disease. The study population consisted of patients who received at least one lipid-lowering medication in 2023 [23].

### 2.2. Lipid-Lowering Therapy Data and Study Parameters

The BDCAP database is organized into distinct analytical modules, each containing specific study variables. For this analysis, data were extracted from the “drugs” module, which records medications actively prescribed and/or dispensed during the study period. The study focused on lipid-lowering therapies classified under the Anatomical Therapeutic Chemical (ATC) code C10A, which includes commonly used medications such as statins (e.g., simvastatin, atorvastatin), fibrates, bile acid sequestrants, and other lipid-modifying agents, analyzing prescribing patterns in patients undergoing secondary prevention. Secondary prevention patients were identified based on diagnostic codes from the International Classification of Primary Care, version 2 (ICPC-2), specifically including individuals with a recorded history of ischemic heart disease with angina (K74), acute myocardial infarction (K75), ischemic heart disease without angina (K76), transient cerebral ischemia (K89), stroke (K90), cerebrovascular disease (K91), or peripheral arterial disease/atherosclerosis (K92). Key variables included the prevalence of lipid-lowering drug prescriptions, stratified by gender (male and female) and age, using predefined age groups: <40 years, 40–44, 45–49, 50–54, 55–59, 60–64, 65–69, 70–74, 75–79, 80–84, and ≥85 years. Socioeconomic factors such as municipality size (<10,000, 10,001–50,000, 50,001–100,000, 100,001–500,000, and >500,000 inhabitants), income level (categorized as ≥100,000 €/year, 18,000–99,999 €/year, <18,000 €/year, and “very low”)**,** and employment status (employed, unemployed, inactive, retired, or other) were also considered to explore disparities in treatment patterns. Additionally, treatment type was categorized into single-drug therapy and combination therapy (i.e., the use of two or more lipid-lowering medications) to assess trends in the adoption of combination regimens, which are increasingly relevant for achieving stricter lipid control. Temporal trends were examined over a 5-year period (2019–2023), evaluating the prevalence of lipid-lowering therapy use, as well as the defined daily dose (DDD) per 1000 individuals per day. The DDD, established by the World Health Organization (WHO), refers to the assumed average maintenance dose per day of a drug used for its primary indication in adults. This standardized unit allows for meaningful comparisons of medication use across populations and time periods. The analysis also assessed the proportion of patients achieving LDL-C serum levels below 70 mg/dL and below 100 mg/dL, comparing the effectiveness of monotherapy versus combination therapy [14,21]. A regional analysis was also conducted to identify geographic variations in lipid-lowering treatment utilization across Spain’s Autonomous Communities, providing a comprehensive assessment of treatment patterns and therapeutic effectiveness in secondary prevention.

### 2.3. Data Analysis

Descriptive statistics were used to summarize the study population, expressing quantitative variables as absolute values and relative percentages concerning the total sample or specific subpopulations. Age- and gender-stratified results were standardized per 1000 individuals attended in the SHS to facilitate comparability. Categorical variables, such as gender, employment status, and treatment type, were analyzed using chi-square tests, while continuous variables were assessed using *t*-tests or the Mann–Whitney U test, depending on data distribution. In addition to descriptive analyses, chi-square tests were used to assess differences in lipid-lowering therapy use across regions, treatment types (monotherapy vs. combination), and by type of vascular disease (ischemic heart disease, stroke, or peripheral artery disease). Chi-square tests were also applied to compare the distribution of LDL-C intervals between treatment groups. Linear trend analyses were performed using linear regression models to evaluate changes over time in the prevalence of treatment use and in the proportion of patients achieving LDL-C targets (<70 mg/dL and <100 mg/dL), stratified by sex. Statistical significance was set at *p* < 0.05 for all comparisons. Temporal trends in lipid-lowering therapy use, distinguishing between monotherapy and combination therapy, were evaluated from 2019 to 2023, along with the evolution of DDD per 1000 individuals per day. Additionally, subgroup analyses assessed LDL-C outcomes, focusing on the proportion of patients achieving target LDL-C levels below 70 mg/dL and below 100 mg/dL, stratified by treatment type. LDL-C levels were reported in the BDCAP database only as categorical variables using the thresholds < 70 mg/dL and <100 mg/dL. More stringent cut-offs recommended by the 2019 ESC/EAS Guidelines (<55 mg/dL for very high-risk patients and <40 mg/dL for those with recurrent events within 2 years) were not available in the dataset, representing a limitation of the present analysis. A regional analysis was also conducted to examine geographic variations in treatment prevalence and combination therapy use across Spain’s Autonomous Communities, providing a comprehensive evaluation of lipid-lowering therapy utilization and effectiveness in secondary prevention. All statistical analyses were performed using R software (version 4.2.1; Austria) [24].

## 3. Results

**Figure 1:**
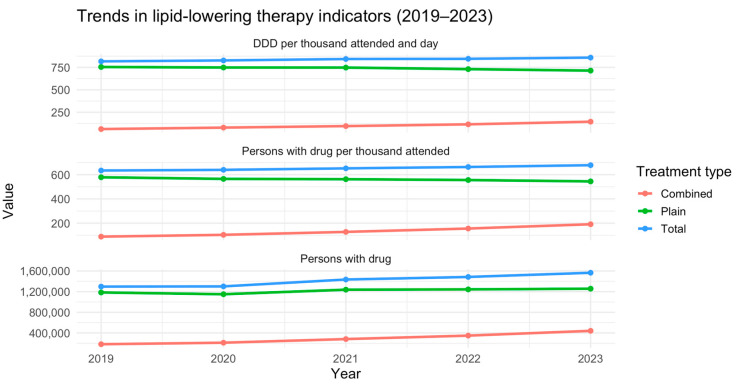
Type of lipid-lowering drugs taken by year (2019–2023) in secondary prevention. DDD: Defined Daily Dose refers to the assumed average maintenance dose per day of a drug used for its primary indication in adults. Note: The sum of individuals receiving plain and combination therapy does not equal the total number of treated patients, as individuals may switch between treatment groups over the course of the year.

In 2023, a total of 7.3 million individuals in Spain received at least one lipid-lowering medication, according to BDCAP data. Of these, 1,565,429 patients (21.4%) were classified as receiving treatment for secondary prevention, corresponding to 678.3 per 1000 individuals attended in the SHS with a prior diagnosis of ischemic heart disease, stroke, or peripheral artery disease. The remaining 5.8 million patients were treated for primary prevention. Among secondary prevention patients, 1,256,901 individuals (80.3%) received monotherapy, while 440,879 (19.7%) were prescribed a combination of lipid-lowering drugs (Figure 1). Stratified by sex, 743.9 per 1000 men and 605.1 per 1000 women received lipid-lowering therapy for secondary prevention. As expected, treatment use was more common among retired individuals, while no significant differences were found based on income level or municipality size (Table 1).

**Figure 2:**
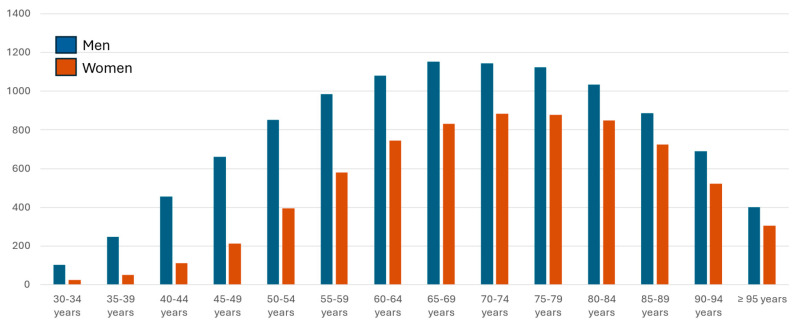
DDD per thousand persons attended per day in secondary prevention.

Age-group analysis shows that most treated patients are over 65 years old, for both men and women. The number of patients receiving treatment increases with age until 85 years, after which it declines (Table 2, Figure 2).

**Figure 3:**
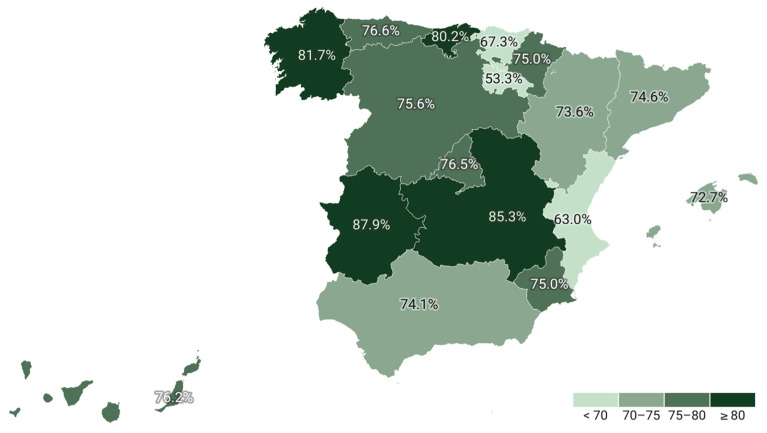
Percentage of persons with lipid lowering drugs in secondary prevention by region.

**Figure 4:**
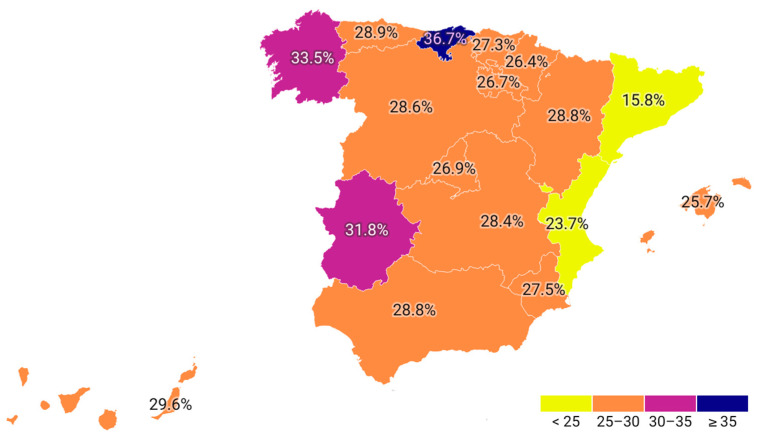
Percentage of persons with combined lipid lowering drugs in secondary prevention by region.

Regional analysis across the Spanish Autonomous Communities reveals notable disparities. The proportion of treated individuals ranged from 53.9% to 87.9% of patients attended, while the percentage of those receiving combination therapy varied between 15.7% and 36.7% (Table 3, Figure 3 and Figure 4). Chi-square analyses confirmed that these differences between regions were statistically significant for both the overall treatment rate (*p* < 0.001) and the use of combination lipid-lowering therapy (*p* < 0.001). Regarding the type of prior vascular event, 1,153,884 patients had a history of ischemic heart disease, 901,745 had experienced a stroke, and 615,238 had peripheral artery disease, with possible overlap across categories (Table 4). Ischemic heart disease was the most prevalent condition, representing the group most likely to receive both lipid-lowering therapy overall and combination therapy specifically. The temporal analysis reveals a steady increase in the number of patients receiving lipid-lowering treatment over recent years, accompanied by an increase in the defined daily dose. While the proportion of patients using monotherapy has declined (*p* = 0.08), the use of combination therapy has risen from 88.9 per 1000 attended individuals in 2019 to 191.1 per 1000 in 2023 (*p* = 0.002) (Figure 1).

**Figure 5:**
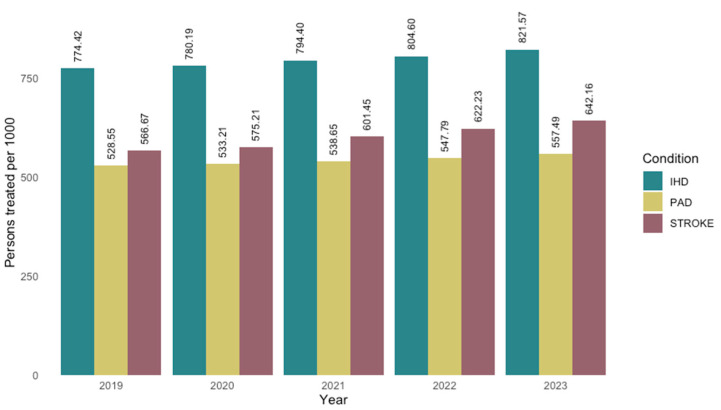
Persons treated per thousand persons attended in secondary prevention by type of previous disease and year. PAD: Peripheral Artery Disease; IHD Ischemic Heart Disease.

**Figure 6:**
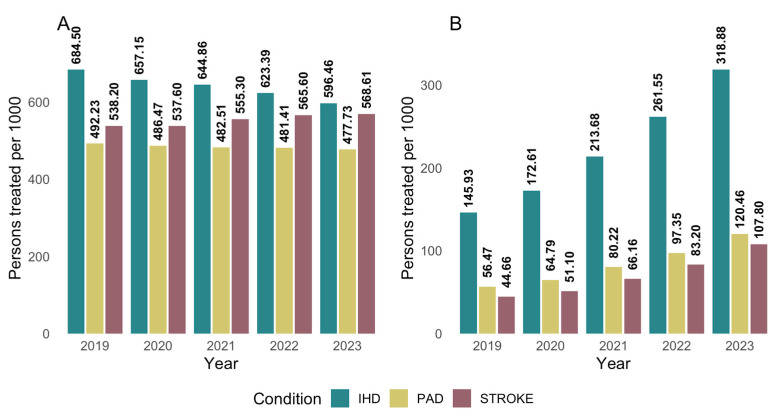
Persons treated with plain (**A**) and combined treatment (**B**) per thousand persons attended in secondary prevention by type of previous disease and year. PAD: Peripheral Artery Disease; IHD Ischemic Heart Disease.

When analyzing patients by vascular pathology, those diagnosed with ischemic heart disease were the most likely to be receiving lipid-lowering therapy (82.1% in 2023), followed by patients with a history of stroke (64.2%) and those diagnosed with peripheral artery disease (56.7%) (Table 4, Figure 5). Patients with ischemic heart disease were also more likely to receive combination lipid-lowering therapy (Figure 6). However, an increasing trend in combination therapy use was observed across all patient groups. Chi-square analyses confirmed that these differences in lipid-lowering drug use by vascular disease subtype were statistically significant, both in overall treatment rates and in the use of combination therapy (*p* < 0.001).

**Figure 7:**
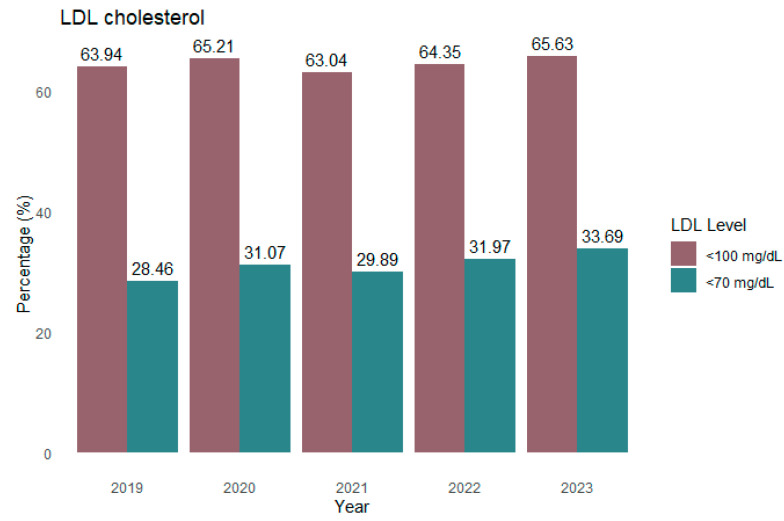
Levels of LDL cholesterol achieved by year.

**Figure 8:**
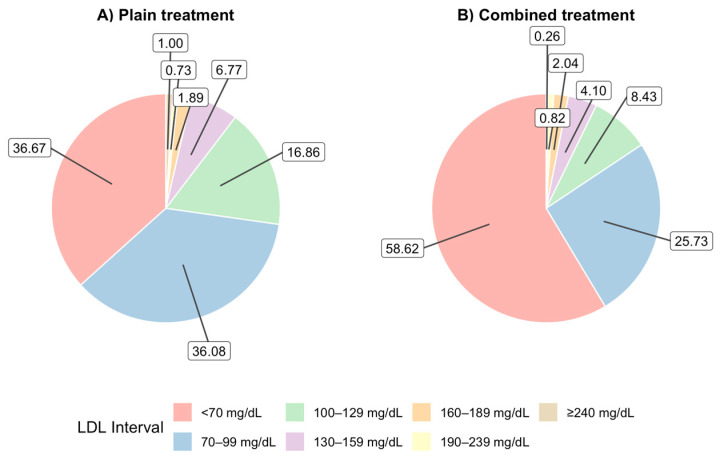
Intervals of LDL cholesterol achieved and type of lipid-lowering treatment.

Regarding serum LDL cholesterol levels in treated patients, only 33.7% achieved levels below 70 mg/dL in 2023, while 65.6% reached levels below 100 mg/dL (Figure 7). A significantly higher proportion of patients on combination therapy achieved LDL-C levels below 70 mg/dL (58.6% vs. 36.7%) and below 100 mg/dL (84.3% vs. 72.8%) compared to those on monotherapy (Figure 8). Chi-square analysis confirmed that the distribution of LDL-C levels differed significantly between treatment types, with patients on combination therapy showing a more favorable lipid profile (*p* < 0.001) (Figure 8).

**Figure 9:**
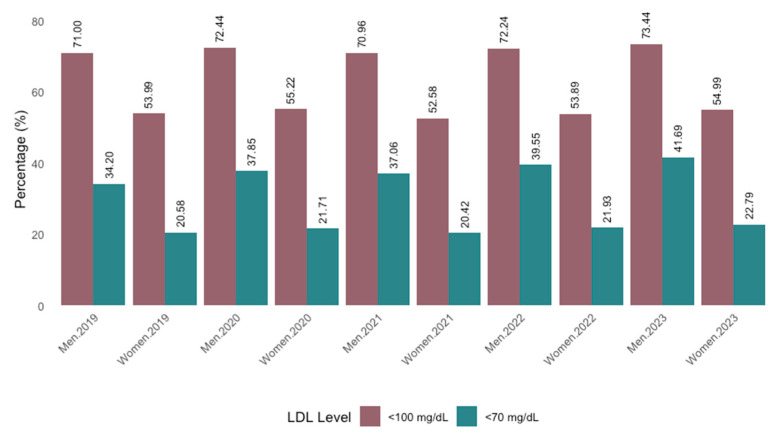
Levels of LDL cholesterol achieved by year and sex.

The percentage of patients achieving LDL-C levels below 70 mg/dL or 100 mg/dL has progressively increased over recent years, although levels remain consistently lower in women than in men. This gender difference has persisted over time (Figure 9). Trend analyses revealed a statistically significant increase in the proportion of men achieving LDL-C levels below 70 mg/dL between 2019 and 2023 (*p* = 0.016); however, no significant trends were observed in women, nor for LDL-C levels below 100 mg/dL in either sex (*p* > 0.15 for all) (Figure 9).

## 4. Discussion

This study offers a thorough evaluation of lipid profile management in secondary prevention patients throughout Spain, drawing upon data from the BDCAP registry. In 2023, 1,565,429 patients (678.3 per 1000 attended) with established ischemic heart disease, stroke, or peripheral artery disease received lipid-lowering therapy. Only 33.7% of treated patients achieved LDL-C levels below 70 mg/dL, while 65.6% reached levels below 100 mg/dL, with combination therapy showing clear superiority over monotherapy in achieving both targets. Marked gender differences were observed, with treatment rates of 743.9 per 1000 in men compared to 605.1 per 1000 in women. Regional disparities were also notable, with treatment coverage ranging from 53.9% to 87.9% and combination therapy use from 15.7% to 36.7%. The proportion of patients on combination therapy increased steadily from 88.9 per 1000 in 2019 to 191.1 per 1000 in 2023.

**LDL-C Goal Attainment**

These findings reveal persistent gaps in achieving LDL cholesterol (LDL-C) targets, despite a notable increase in the use of lipid-lowering therapies, particularly combination regimens. These results underscore the critical need for the development and implementation of more effective lipid management strategies to optimize cardiovascular risk reduction in this high-risk population. While current ESC and SEC guidelines recommend achieving LDL-C levels below 70 mg/dL in secondary prevention, this goal is often difficult to achieve in routine practice [5,6]. Barriers include suboptimal treatment adherence, statin intolerance, limited access to PCSK9 inhibitors, and variability in guideline implementation between regions [15,25]. Furthermore, the ‘lipid paradox’—a phenomenon in which lower cholesterol levels have been associated with higher mortality in certain populations, such as elderly or frail patients, possibly due to reverse causality or comorbidities—should be considered when interpreting our results [26,27]. Although this paradox does not negate the strong evidence supporting LDL-C reduction for cardiovascular risk lowering, it underscores the need to individualize treatment targets, particularly in patients with advanced age, multiple comorbidities, or poor nutritional status. In our cohort, the lack of patient-level data on frailty and comorbid conditions limits our ability to fully explore this effect, but the potential influence of these factors should be acknowledged when extrapolating our conclusions.

**Treatment Patterns: Monotherapy vs. Combination Therapy**

The findings of the present analysis demonstrate that, in 2023, only 33.7% of treated patients achieved LDL-C levels below 70 mg/dL, while 65.6% reached levels below 100 mg/dL. These findings are consistent with the findings of previous studies that reported suboptimal LDL-C control among secondary prevention populations, where achievement rates for more stringent targets (e.g., 70 mg/dL) typically range between 33% and 35% [5,28]. Notably, combination therapy demonstrated a marked superiority over monotherapy in achieving both LDL-C targets, underscoring its role as a preferred strategy for patients requiring intensive lipid-lowering interventions [23]. When considering whether two older-generation lipid-lowering drugs could be more effective than a single newer agent, current evidence indicates that the choice should be individualized according to the patient’s lipid profile, residual cardiovascular risk, and tolerability. For example, adding a fibrate or bile acid sequestrant to a statin may provide additional benefit in patients with mixed dyslipidemia or persistent hypertriglyceridemia [5,29]; however, in patients with markedly elevated LDL-C despite maximally tolerated statin therapy, the addition of ezetimibe or a PCSK9 inhibitor offers greater LDL-C reduction and stronger evidence for cardiovascular event reduction [15,25,30,31]. Head-to-head comparisons suggest that combination therapy with moderate- or high-intensity statins plus ezetimibe achieves LDL-C reductions comparable to high-intensity statin monotherapy, with better tolerability [10,11]. Therefore, while older drug combinations may be appropriate in specific contexts, guideline-directed combinations involving newer agents are generally more effective in achieving stringent LDL-C goals and improving long-term outcomes.

**Other Cardioprotective Therapies**

In addition to lipid-lowering therapy, many secondary prevention patients are concurrently treated with other pharmacological agents with established cardiovascular benefits, which may influence lipid management decisions and outcomes. For example, glucagon-like peptide-1 receptor agonists (GLP-1 RAs) have demonstrated significant cardiovascular risk reduction in patients with obesity and type 2 diabetes, independent of their effects on lipid levels [32,33]. Similarly, angiotensin-converting enzyme inhibitors (ACEIs) and angiotensin receptor blockers (ARBs) are widely used in patients with hypertension, heart failure, or post-myocardial infarction, and have been shown to improve cardiovascular prognosis [34,35]. In clinical practice, the presence of multiple cardioprotective therapies may lead clinicians to prioritize comprehensive cardiovascular risk management over the further intensification of lipid-lowering therapy, particularly in patients who are already achieving multifactorial risk reduction. This therapeutic overlap could partially explain the suboptimal LDL-C goal attainment observed in our cohort, as additional lipid-lowering agents might be deferred when patients are already on complex medication regimens.

**Differences by Vascular Pathology**

An important stratification by vascular pathology revealed that patients diagnosed with ischemic heart disease were most likely to receive lipid-lowering therapy (82.1% in 2023), followed by those with a history of stroke (64.2%) and peripheral artery disease (56.7%) [36]. Moreover, patients with ischemic heart disease were more likely to receive combination lipid-lowering therapy, reflecting a tailored approach to managing their elevated risk. This trend may be attributed to cardiologists’ heightened awareness and adherence to guideline-recommended lipid-lowering treatments for patients with ischemic heart disease. Studies have shown that patients under cardiology care are more likely to be prescribed lipid-lowering medications compared to those managed solely in primary care settings. Additionally, the proportion of patients with stroke who received high-intensity statin therapy was significantly higher when discharged from cardiology wards compared to neurology wards, suggesting a specialty-based variation in prescribing practices [37,38].

**Gender Disparities**

When gender differences were considered, the study revealed that lipid-lowering drugs were prescribed at a rate of 743.9 per 1000 men and 605.1 per 1000 women [39]. While this discrepancy might initially suggest gender differences in cardiovascular disease prevalence or prescribing patterns, in our study, all patients, both men and women, had established cardiovascular disease (100% prevalence). This means the lower rate of lipid-lowering therapy in women cannot be explained by prevalence differences. Instead, it likely reflects other factors not explored here, such as reduced likelihood of being offered statins, higher rates of statin side effects and discontinuation, and gender bias in treatment decisions. For example, the PALM registry found that women were significantly less likely than men to be prescribed any statin (67.0% vs. 78.4%) or at the guideline-recommended intensity (36.7% vs. 45.2%); they were also more inclined to decline statins or discontinue them due to side effects [12]. Additionally, real-world analyses confirm that women on statins report greater intolerance and non-adherence due to side effects, while other studies highlight persistent gender bias leading to less aggressive secondary prevention care in women [16].

**Regional Differences and Age Patterns**

Regional disparities were also evident, with treatment coverage ranging from 53.9% to 87.9% and combination therapy utilization varying between 15.7% and 36.7%. These disparities may be attributable to variations in healthcare delivery systems, adherence to clinical guidelines, or resource allocation across Spain’s Autonomous Communities [10]. Moreover, an analysis of age demographics indicates that lipid-lowering therapy is most utilized among individuals over the age of 65, with a subsequent decline after the age of 85 [11,40,41].

**Implications and Related Evidence**

The observed LDL-C control rates are consistent with findings from European studies, such as DYSIS-Spain, which reported that only a minority of high-risk patients achieved recommended lipid targets [13]. Consistent with these findings, studies conducted in other European populations have documented achievement rates below 50% for LDL-C levels 70 mg/dL, particularly among older adults and those with recurrent cardiovascular events [42,43,44]. However, our study provides novel insights by demonstrating temporal improvements in LDL-C target attainment over recent years. These improvements are likely driven by the increasing adoption of combination therapy and targeted approaches based on vascular pathology. In addition to the management of LDL-C, the treatment of atherogenic dyslipidemia, defined by elevated levels of triglycerides and apolipoprotein B, has garnered attention due to the increasing prevalence of obesity and insulin resistance [15]. This lipid profile is becoming increasingly prevalent and requires additional therapeutic interventions beyond traditional statin-based regimens.

**Clinical Recommendations**

The low proportion of patients achieving LDL-C targets underscores the necessity for more aggressive lipid-lowering strategies and improved adherence to evidence-based guidelines [25]. Combination therapies should be prioritized for patients with established atherosclerotic cardiovascular disease, such as ischemic heart disease, who fail to reach LDL-C targets with statins alone. Although LDL-C-lowering goals (<70 mg/dL or even <55 mg/dL) are shared across high-risk conditions such as stroke and peripheral artery disease, evidence shows that patients with recent acute coronary syndrome benefit particularly from early, aggressive combination lipid lowering. The IMPROVE-IT trial demonstrated that adding ezetimibe to simvastatin in post-ACS patients significantly decreased recurrent major cardiovascular events compared to statin monotherapy [30,45]. Furthermore, contemporary analyses indicate that moderate-intensity statin plus ezetimibe achieves LDL-C reductions comparable to high-intensity statin alone, with improved adherence and lower LDL-C variability—crucial for stable, durable lipid control in IHD [31]. These findings support current ESC and ACC guidelines that recommend upfront combination therapy in very high-risk patients, particularly after an ACS event, to rapidly and reliably achieve LDL-C targets and reduce residual cardiovascular risk [29,46]. Furthermore, non-pharmacological interventions, such as dietary modifications (e.g., adherence to a Mediterranean diet), increased physical activity, and smoking cessation programs, should be integrated into comprehensive care plans [47].

The observed gender disparities in the utilization of lipid-lowering therapy underscore the necessity for further investigation into potential discrepancies in treatment access and outcomes. By elucidating the factors contributing to these disparities, we can develop more targeted and equitable approaches to cardiovascular risk management. The observed regional disparities in treatment coverage and combination therapy use suggest the presence of inequities in healthcare delivery [48], which necessitates further investigation. Addressing these disparities has the potential to markedly improve cardiovascular outcomes throughout Spain.

**Limitations and Strengths**

The main limitation of this study is the inability to assess the proportion of patients who achieved LDL-C levels below 55 mg/dL, which is the recommended target for patients at very high cardiovascular risk according to current European guidelines [1,5]. This means that the reported attainment rates for <70 mg/dL almost certainly overestimate the proportion of patients meeting the actual recommended targets of <55 mg/dL or <40 mg/dL. Due to data availability constraints, we were only able to analyze achievement rates for LDL-C thresholds of <70 mg/dL and <100 mg/dL. Other limitations must also be considered. The cross-sectional design precludes causal inference regarding the effectiveness of lipid-lowering therapies. The BDCAP database, being administrative in nature, depends on the quality and completeness of data recorded by each Autonomous Community’s information systems. The absence of information on the proportion of missing data per variable may affect the interpretation of regional disparities and warrants caution. Importantly, clinical variables that could define individual cardiovascular risk, such as lipid profiles at baseline, smoking status, or family history, are not available, limiting the depth of analysis on sex- and age-related prescribing differences. The database does not include adherence data, which may have influenced observed LDL-C outcomes, nor does it allow exploration of broader pharmacological profiles or non-lipid cardiovascular risk management. Furthermore, the BDCAP database does not include baseline LDL-C values, serial lipid measurements, or treatment sequence data. As such, we could not quantify the proportion of patients with a clear indication for combination lipid-lowering therapy, such as those with very high baseline LDL-C or persistent elevation despite statin monotherapy, who were nevertheless treated only with a single statin. Given prior real-world studies, this proportion is likely substantial, representing an important missed opportunity for cardiovascular risk reduction. Socioeconomic variables, including income and education, were also not fully assessed, restricting the ability to investigate potential inequalities in access to, or quality of, care. Finally, although the study focused on secondary prevention, comparisons with primary prevention groups could provide useful context and highlight further gaps in cardiovascular risk management.

A key strength of this study lies in its large sample size and the use of a comprehensive national dataset (BDCAP), which ensures a high degree of representativeness for the Spanish population. The nature of the data enables the analysis of temporal trends in lipid-lowering therapy use and LDL-C target attainment over a 5-year period, offering valuable insights into evolving clinical practices across diverse patient subgroups. Additionally, the structured coding system and stratified data allow for meaningful analyses by region, age, sex, and vascular pathology.

Further research is needed to identify barriers to achieving LDL-C targets in secondary prevention patients, including factors related to healthcare delivery systems and patient adherence [48]. Furthermore, the exploration of advanced technologies, such as telemedicine and personalized medicine, holds promise in enhancing patient outcomes by promoting adherence and facilitating customized treatment approaches.

## 5. Conclusions

In summary, the present study underscores substantial deficiencies in lipid management among secondary prevention patients in Spain, notwithstanding the escalating utilization of combination therapies. To enhance cardiovascular risk reduction in this high-risk population, there is an imperative need to implement the following: (1) improve adherence to clinical guidelines; (2) address regional disparities; and (3) consider gender-specific differences. Future strategies should prioritize the integration of pharmacological and non-pharmacological interventions to achieve more comprehensive lipid control.

## Figures and Tables

**Table 1:** Patients taking lipid-lowering drugs in 2023 in secondary prevention.

	DDD per Thousand Attended and Day	Total Number	Per Thousand Attended	*p*-Value
**Total**	859.15	1,565,429	678.35	
**Gender**				
Women	686.02	560,660	605.09	
Men	997.46	1,004,511	743.92	<0.01
**Income level**				
≥100,000 €/year	854.95	12,605	705.84	
18,000–99,999 €/year	875.5	521,752	694.62	
<18,000 €/year	830.31	586,555	656.62	
Very low	886.88	441,355	692.86	
**Size of the municipality**				
<10,000 inhab.	878.36	249,776	682.22	
<10,000 inhab.	860.39	480,023	670.94	
50,001–100,000 inhab.	839.44	216,984	677.81	
100,001–500,000 inhab.	860.39	381,260	683.87	
>500,000 inhab.	852.53	237,386	681.19	
**Employment status**				
Employed	603.36	186,920	509.22	
Unemployed	699.12	46,444	574.36	
Inactive	626.52	110,471	547.61	
Retired	973.92	1,154,475	751.74	<0.001
Other	675.17	67,119	548.84	

DDD: Defined Daily Dose refers to the assumed average maintenance dose per day of a drug used for its primary indication in adults.

**Table 2:** People taking lipid lowering drugs by age and sex in secondary prevention (2023).

	Men			Women			Total		
	DDD per thousand attended and day	Total number	Per thousand attended	DDD per thousand attended and day	Total number	Per thousand attended	DDD per thousand attended and day	Total number	Per thousand attended
**30–34 years**	102.61	708	109.08	24.82	363	30.71	52.38	1072	58.47
**35–39 years**	248.37	2174	236.58	51.74	942	60.51	124.74	3116	125.88
**40–44 years**	454.12	7255	397.44	112.4	3041	128.79	261.4	10,297	245.93
**45–49 years**	661.05	21,282	54.8	211.82	7401	214.82	449.7	28,683	391.68
**50–54 years**	850.77	45,746	664.48	395.23	16,677	376.43	672.4	62,423	551.69
**55–59 years**	983.36	85,343	736.8	579.52	31,365	527.72	846.41	116,707	665.9
**60–64 years**	1080.90	126,585	782.16	743.3	48,768	645.87	973.5	175,353	738.8
**65–69 years**	1152.30	153,124	818.96	830.04	64,412	712.59	1047.28	217,536	784.3
**70–74 years**	1144.82	163,672	824.54	882.2	78,573	744.68	1053.67	242,245	796.82
**75–79 years**	1124.07	167,424	820.32	877.37	97,789	749.61	1027.87	265,214	792.74
**80–84 years**	1034.25	121,039	789	848.34	88,905	724.92	951.65	209,944	760.53
**85–89 years**	886.52	76,188	716.26	725.69	76,132	652.08	802.36	152,320	682.68
**90–94 years**	690.25	29,430	608.29	522.04	38,317	523.43	588.98	67,746	557.2
**≥95 years**	400.48	4232	435.04	304.32	7974	348.15	332.99	12,206	374.06
**Total**	1015.04	1,004,203	756.92	686.02	560,660	605.09	879.75	1,564,863	694.49

DDD: Defined Daily Dose refers to the assumed average maintenance dose per day of a drug used for its primary indication in adults.

**Table 3:** Lipid lowering drugs by region in 2023 in secondary prevention.

	Population Attended	Persons with Drug	Percentage with Drug	Plain Drug	Combined Drug	Percentage with Combined Drugs over Total Taking Lipid Lowering Drugs
**Andalucía**	396,924	294,090	74.09%	209,273	84,817	28.84%
**Aragón**	60,418	44,478	73.62%	31,654	12,824	28.83%
**Asturias**	72,131	55,247	76.59%	39,301	15,946	28.86%
**Baleares**	57,703	41,939	72.68%	31,148	10,791	25.73%
**Canarias**	117,455	89,538	76.23%	63,030	26,508	29.61%
**Cantabria**	31,454	25,209	80.15%	15,958	9251	36.70%
**Castilla y León**	134,884	102,020	75.64%	72,832	29,188	28.61%
**Castilla la Mancha**	33,411	28,510	85.33%	20,420	8090	28.38%
**Cataluña**	397,504	296,588	74.61%	249,864	46,724	15.75%
**Comunidad Valenciana**	321,402	202,441	62.99%	154,378	48,063	23.74%
**Extremadura**	30,783	27,071	87.94%	18,476	8595	31.75%
**Galicia**	142,255	116,185	81.67%	77,263	38,922	33.50%
**Madrid**	274,895	210,195	76.46%	153,689	56,506	26.88%
**Murcia**	64,694	48,529	75.01%	35,162	13,367	27.54%
**Navarra**	34,285	25,160	73.38%	18,518	6642	26.40%
**País Vasco**	123,358	83,051	67.33%	60,414	22,637	27.26%
**La Rioja**	14,135	7531	53.28%	5522	2009	26.68%
**Total**	**2,307,689**	**1,697,780**	**73.57%**	**1,256,901**	**440,879**	**25.97%**

**Table 4:** Lipid-lowering drugs taken in secondary prevention by type of previous event in 2023.

		2023		
		PAD	Stroke	IHD
**Plain drug**	DDD per thousand attended and day	543.67	568.61	596.46
	Persons with drug per thousand attended	477.73	568.61	596.46
**Combined**	DDD per thousand attended and day	89.7	77.77	246.55
	Persons with drug per thousand attended	120.46	107.84	318.88
**Total**	DDD per thousand attended and day	633.37	822.32	1119.62
	Persons with drug per thousand attended	557.49	642.16	821.57

DDD: Defined Daily Dose refers to the assumed average maintenance dose per day of a drug used for its primary indication in adults. PAD: Peripheral Artery Disease; IHD: Ischemic Heart Disease.

## Data Availability

All data generated in this study are available from the corresponding authors upon reasonable request. Additionally, some data can be accessed through the following website: https://www.sanidad.gob.es/estadEstudios/estadisticas/estadisticas/estMinisterio/SIAP/home.htm (accessed on 29 March 2025).
